# NADPH and Mito-Apocynin Treatment Protects Against KA-Induced Excitotoxic Injury Through Autophagy Pathway

**DOI:** 10.3389/fcell.2021.612554

**Published:** 2021-02-11

**Authors:** Na Liu, Miao-Miao Lin, Si-Si Huang, Zi-Qi Liu, Jun-Chao Wu, Zhong-Qin Liang, Zheng-Hong Qin, Yan Wang

**Affiliations:** Department of Pharmacology and Laboratory of Aging and Nervous Diseases and Jiangsu Key Laboratory of Neuropsychiatric Diseases, College of Pharmaceutical Sciences, Soochow University, Suzhou, China

**Keywords:** *e*xcitotoxicity, ROS, NADPH, Mito-apocynin, NOX, *m*itochondria, autophagy

## Abstract

**Aim:**

Previous research recognizes that NADPH can produce reduced glutathione (GSH) as a coenzyme and produce ROS as a substrate of NADPH oxidase (NOX). Besides, excessive activation of glutamate receptors results in mitochondrial impairment. The study aims at spelling out the effects of NADPH and Mito-apocynin, a NOX inhibitor which specifically targets the mitochondria, on the excitotoxicity induced by Kainic acid (KA) and its mechanism.

**Methods:**

The *in vivo* neuronal excitotoxicity model was constructed by stereotypically injecting KA into the unilateral striatum of mice. Administrated NADPH (*i.v*, intravenous) 30 min prior and Mito-apocynin (*i.g*, intragastric) 1 day prior, respectively, then kept administrating daily until mice were sacrificed 14 days later. Nissl staining measured the lesion of striatum and survival status of neurons. Cylinder test of forelimb asymmetry and the adhesive removal test reflected the behavioral deficit caused by neural dysfunction. Determined Total superoxide dismutase (T-SOD), malondialdehyde (MDA), and GSH indicated oxidative stress. Western blot presented the expression levels of LC3-II/LC3-I, SQSTM1/p62, TIGAR, and NOX4. Assessed oxygen consumption rate using High-Resolution Respirometry. *In vitro*, the MitoSOX Indicator reflected superoxide released by neuron mitochondria. JC-1 and ATP assay Kit were used to detect mitochondrial membrane potential (MMP) and energy metabolism, respectively.

**Results:**

In this study, we have successfully established excitotoxic model by KA *in vivo* and *in vitro*. KA induced decreased SOD activity and increased MDA concentration. KA cause the change of LC3-II/LC3-I, SQSTM1/p62, and TIGAR expression, indicating the autophagy activation. NADPH plays a protective role *in vivo* and *in vitro*. It reversed the KA-mediated changes in LC3, SQSTM1/p62, TIGAR, and NOX4 protein expression. Mito-apocynin inhibited KA-induced increases in mitochondrial NOX4 expression and activity. Compared with NADPH, the combination showed more significant neuroprotective effects, presenting more neurons survive and better motor function recovery. The combination also better inhibited the over-activated autophagy. *In vitro*, combination of NADPH and Mito-apocynin performed better in restoring mitochondria membrane potential.

**Conclusion:**

In summary, combined administration of NADPH and NOX inhibitors offers better neuroprotection by reducing NADPH as a NOX substrate to generate ROS. The combined use of NADPH and Mito-apocynin can better restore neurons and mitochondrial function through autophagy pathway.

## Introduction

Glutamate is an important excitatory neurotransmitter in the central nervous system and plays a crucial role in a variety of neural processes including cognition, learning and memory (Barker-Haliski and White, 2015; Reiner and Levitz, 2018). However, under pathophysiology, excessive release of glutamate can lead to over-activation of glutamate receptors, leading to death of neurons (Wang and Qin, 2010). Excitotoxicity induced by overstimulation of glutamate receptors can be observed in varied brain diseases, such as ischemia, stroke, epilepsy, and neurodegenerative diseases (Dong et al., 2009; Lai et al., 2014). It is related to free radicals produced by activation of calcium-dependent enzymes, nitric oxide synthase, xanthine oxidase and mitochondrial oxidative dysfunction, and reduce brain antioxidant enzymes, such as glutathione (GSH) peroxidase, superoxide dismutase (SOD) and catalase, lead to the development of neurodegenerative diseases (Rueda et al., 2016). Therefore, if not balanced by endogenous antioxidant mechanisms, ROS increase may pose a potential threat to intracellular homeostasis and neuronal survival.

In our previous studies, NADPH supplementation significantly increased TIGAR expression, inhibited ROS levels and autophagy/lysosomal pathways, and thus protected neurons from KA-induced excitotoxicity *in vivo* and *in vitro* (Liu et al., 2020). However, the therapeutic dose window for NADPH is narrow, so thinking about strategies is necessary for its clinical application (Huang et al., 2018). NADPH has two biological roles in the regulation of redox: the first is as a pivotal component of cellular antioxidant system; the second is to act as a substrate for NADPH oxidase (NOX), which plays a key role in many biological and pathological processes by producing ROS. NOX1, NOX2, and NOX4 are the major subtypes of NOX in the central system that play a major role in brain injury and neurodegenerative diseases. They can produce superoxide anion or hydrogen peroxide from NADPH as substrate (Liu et al., 2020). Therefore, it is necessary to investigate whether NOX inhibitors such as apocynin can be used in combination with NADPH to better protect neurons from KA-induced excitotoxicity.

In addition, mitochondria in the central nervous system are often thought to be the source of ROS in brain aging and age-related neurodegenerative diseases (Wang et al., 2019). Recent evidence suggests that isotypes of enzymes such as isocitrate dehydrogenase (IDH), malic enzyme (ME), aldehyde dehydrogenase (ALDH), and methylene tetrahydrofolate dehydrogenase (MTHFD) catalyze similar reactions of NADPH regeneration in both of cytoplasm and mitochondria and may transfer reduction equivalents between mitochondria and cytoplasm (Lewis et al., 2014; Xu et al., 2018; Bradshaw, 2019). Data have shown that NADPH plays an important role in mitochondrial oxidative damage and the protection of mitochondrial DNA integrity (Ying, 2008). At the same time, NOX enzymes (especially NOX4) are expressed in mitochondria, and the mitochondrial respiratory chain may be the victim rather than the source of ROS production in the cell. NOX can interact with mitochondrial complex 1 and inhibit its activity, and meanwhile regulate mitochondrial biogenesis and energy generation through Nrf2 pathway (Dinkova-Kostova and Abramov, 2015). Therefore, whether exogenous NADPH supplementation and the use of inhibitors targeting mitochondrial NOX can affect mitochondrial redox and mitochondrial function in excitotoxicity remains to be investigated. In this study, Mito-apocynin (apocynin conjugated to the mitochondria-targeting triphenyl phosphonium cation moiety TPP^+^) was applied *in vitro* to verify this hypothesis.

In this study, we investigated whether the combination of NADPH and Mito-apocynin had a better protective effect on neuronal survival and function in the KA-induced excitotoxicity, as well as the effect of exogenous NADPH supplementation and mitochondrial targeted NOX inhibitor on mitochondrial REDOX and function.

## Materials and Methods

### Animal Treatment

SPF grade Institute of Cancer Research (ICR) mice, male, 25–30 *g*, were purchased from Zhaoyan (suzhou) new drug research center. Keep the mice at a constant temperature of 22°C and a humidity of 50–60%. Raise them in a well-ventilated environment and give artificial day and night (12 h/12 h) with freedom of drinking water. The animals were utilized in compliance with the institutional animal healthcare regulations. All animal protocols were approved by the Institutional Animal Care and Use Committee of Soochow University.

After anesthetizing mice with chloral hydrate (400 mg/kg), we infused 0.625 nmol KA into right striatum within 2 min at the following coordinates: 0.8 mm anterior to the bregma, 1.8 mm lateral to the sagittal suture, and 3.5 mm ventral to the pial surface. The volume of all intracranial injections was 1 μL. Administrated NADPH (BT04, BONTAC; *i.v*) and Mito-apocynin (HY-135869, MCE; *i.g*) 30 min and 1 day, respectively.

### Nissl Staining

Coronal sections of the brain of mice were prepared. The brain slices were soaked in nissl staining solution for 20–30 min, and decolorized with 75, 95, and 100% ethanol for 2 min in turn. Permeate with paraformaldehyde for 10 min. The film was sealed and observed under a microscope. Count the number of normal striatum central morphology under 20x magnification. Nissl staining and its quantification was performed as described previously (Liu et al., 2020).

### Measurement of Total SOD Activity and MDA Content

Isolated the striatum tissue after clearing the blood of the animals by cardiac perfusion with PBS. After adding 100 μL SOD sample preparation solution to every 10 mg of tissue, homogenize it on ice. Centrifuge for 3–5 min at 12,000 *g* at 4°C, and then extract the supernatant to be measured. 20 μL sample and 60 μL WST-8/enzyme working solution were added to the 96-well plate. After adding the reaction reagent, mix well. Incubate at 37°C for 30 min. The absorbance was determined at 450 nm. Inhibition percentage = (A_*blank control 1*_–A_*sample*_)/(A_*blank control 1*_–A_*blank control 2*_) × 100%. SOD activity unit = inhibition percentage/(1-inhibition percentage) units. SOD activity unit was converted into U/mg protein according to the protein concentration and dilution ratio of the sample. All operations follow the instructions the test kit (wst-8 method; S0101, Beyotime).

Lyse the striatum tissues of animals with appropriate cell lysate and homogenized. Centrifuge at 12,000 *g* for 5 min at 4°C. Determined protein concentration for subsequent calculation. 0.1 mL sample and 0.2 ml MDA detection working fluid were added into the centrifuge tube. Heat at 100°C for 15 min. Centrifuge at 1,000 *g* room temperature for 10 min. 200 μL microliters of supernatant were added to a 96-well plate and the absorbance was then measured at 532 nm. The MDA content in the sample solution was calculated and converted to μmol/mg according to the protein concentration. All operations follow the instructions the test kit (S0131, Beyotime).

### Separation of Mitochondrial and Cytoplasmic Fractions

After the animals are killed, the striatum tissue is immediately separated and placed on ice. Add about 10 times of mitochondrial separation reagent A solution (add 1% PMSF before use), and homogenate on ice for about 30 times. After homogenization, absorb approximately 50 μL as the total component. Centrifuge the supernatant at 4°C at 11,000 *g* for 10 min for extracting mitochondria. Absorb its supernatant as a cytoplasmic component. Added an appropriate amount of mitochondrial separation reagent to the precipitation, and blow the solution evenly, centrifuge at 4°C at 11,000 *g* for 10 min, remove the supernatant. Add solution A to resuspended, add the resuspended solution to the tube containing 22 and 50% Percoll solution, centrifuge at 4°C at 20,000 *g* for 20 min, and carefully transfer the fraction between the Percoll gradient into the new tube, namely the mitochondrial fraction.

### Western Blot Analysis

Western blot analysis was performed on striatal tissues as described previously (Bradshaw, 2019). We used the following primary antibody: anti-NOX4 (ab109225, abcam), anti-LC3B (NB100-2220, Novus), anti-β-Actin (A5441, Sigma Aldrich), anti-SQSTM1/p62 (P0067, Sigma Aldrich), anti-TIGAR (sc-67273, Santa Cruz), anti-PINK1 (6946s, Cell Signaling), and anti-α-Tublin (NB100-690, Novus).

### Behavioral Tests

Adhesive removal test: Before the experiment begins, the animals adapt to the environment in advance. The adhesive strip (0.2 in^2^) was attached to the mouse’s muzzle (dorsal position). Put the animals into the test cage to move freely, observe and record for 60 s or until strip is removed. The result is the waiting time for strip removal (Hernández-Espinosa et al., 2019).

Cylinder test of forelimb asymmetry: Place mice in cylinder with a height of 20 cm and a diameter of 10 cm. Record the frequency of unilateral and bilateral explorations for 3 min. The proportion of unilateral touch assessed limb asymmetry (Hernández-Espinosa et al., 2019).

### Cell Cultures

On day 18, the cortex was dissected from ICR mouse embryos. Digested for 15 min with 2.5% trypsin at 37°C. Then add DNA enzyme and blow for 3 min. Filter with 400 mesh cell filter. Dilute the cell suspension to about 1 million/mL. Neurons were grown for 6–8 DIV in maintenance media (Neurobasal medium supplemented with 2% B-27, 0.5 μM glutamine, and 100 U/ml penicillin and streptomycin) at 37°C in a 5% CO2 incubator. 50% media was replaced in the fifth day. Incubate neurons for 4 h before KA treatment with neurobasal medium containing NADPH and/or Mito-apocynin (HY-135869, MCE). All animal protocols were approved by the Institutional Animal Care and Use Committee of Soochow University.

### Cell Activity Detection

Primary cortical neurons were inoculated with 96-well plates. The medium was removed and 100 μL of medium containing 10% CCK8 reagent were added to each well. Incubate at 37°C in dark for 3 h. The absorbance at 450 nm was detected. Calculate the relative cellular activity.

### Assessment of Oxygen Consumption Rate Using High-Resolution Respirometry

Mitochondria respiration activity was assessed in striatum by High Resolution Respirometry (OROBOROS Instruments, Innsbruck, Austria). Add 2 ml of striatum tissue homogenate to the chamber and wait for the oxygen flux to stabilize. We measured Oxygen Consumption Rate (OCR) during sequential addition of sample. After adding each substance, wait until the OCR stabilizes before adding the next substance. After a stable oxygen routine flux is achieved, add complex I substrates pyruvate 5 μL (final 5 mM), glutamate 10 μL (final 10 mM), and malate 10 μL (final 2 mM) to obtain the leak of complex I. Then, add 10 μL ADP (final 2.5 mM) to obtain the maximum oxidative phosphorylation value of complex I. After stabilization, add 5 μL Cyt C (final 10 μM) to verify the integrity of the mitochondrial membrane. Add complex II substrate succinate 20 μL (10 mM) to get the maximum oxidative phosphorylation value of complex II. Add uncoupler CCCP 1 μl of per step (final 0.05 μM per step) to obtain the maximal uncoupled respiration. We should wait for the stabilization of oxygen flux between the additions and titrate CCCP until it starts inhibiting the tissue oxygen flux. Add 1 μL complex I inhibitor rotenone (final 0.5 μM) to interrupt the electron transfer system to obtain the maximum electron transfer capacity of complex II. Add 1 μL complex III inhibitor Antimycin A (final 2.5 μM) to obtain the residual non-mitochondrial respiration after stabilization of the oxygen flux. Finally, add complex IV substrates 5 μl ascorbate (final 2 mM) and 5 μL TMPD (final 0.5 mM) to detect the function of complex IV.

### Measurement of Mitochondrial Superoxide, Membrane Potential and ATP Levels

Determine the levels of mitochondrial superoxide, membrane potential and ATP with a MitoSOX Red mitochondrial superoxide indicator (40778ES50, Yeasen), mitochondrial membrane potential (MMP) assay kit with JC-1 (C2006, Beyotime), or ATP assay Kit (S0026, Beyotime) following the manufacturer’s instructions.

Primary cortical neurons were inoculated with 24-well plates. Remove the medium and wash twice with HBSS. Add 0.3 mL MitoSOX working fluid and incubated at 37°C in dark for 20 min. Remove supernatant and wash twice with HBSS. Add 0.3 mL Hoechst reagent and incubated at 37°C in dark for 10 min. Remove supernatant and wash twice with HBSS. Add 0.2 mL HBSS and observe with fluorescence microscope.

Primary cortical neurons were inoculated with 24-well plates. Remove the medium and wash twice with PBS. Add 0.3 mL JC-1 working fluid and incubated at 37°C in dark for 20 min. Remove supernatant and wash twice with pre-cooled JC-1 buffer. Add 0.2 mL medium and observe with fluorescence microscope. The presence of green fluorescence indicates that MMP decreases.

The tissue or cultures was mixed with cracking fluid and homogenized with a glass homogenizer. Centrifuge at 4°C for 5 min at 12,000 *g*. Supernatant was taken for subsequent determination. Add 100 μL ATP test working fluid into the test hole. React at room temperature for 3–5 min. Add 20 μL sample, mix quickly and measure RLU value with luminometer. The ATP concentration was calculated according to the standard curve. The concentration of ATP was converted to nmol/mg based on the protein concentration.

### Statistical Analysis

Compare different groups by one-way analysis of variance (ANOVA) with Newman–Keuls *post hoc* test. All data were expressed as means ± SEM. *p* < 0.05 is considered statistically significant.

## Results

### KA-Induced Excitotoxicity Causes Oxidative Stress and Autophagy Activation, Leading to Neuronal Death in the Striatum

**FIGURE 1:**
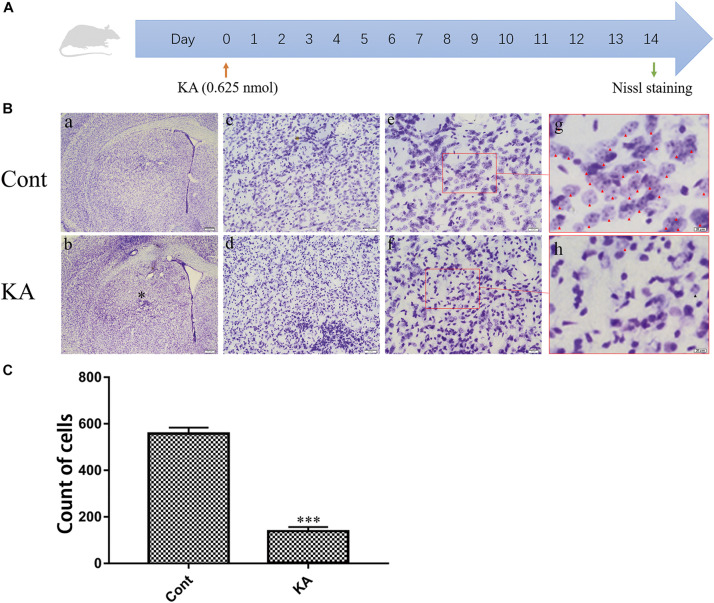
KA causes neuronal injury in the striatum. **(A)** Illustration of experimental design. Mice were sacrificed on day 14 after KA injection. Nissl staining were performed on brain sections. **(B)** Representative microscopic images of Nissl-stained coronal brain section displaying the lesion. **(C)** Quantification of neurons with normal morphological specificity. The histological analyses were single-blinded. Data are expressed as the Mean ± SEM. Scale bar = 500 μm in a, b; 100 μm in c, d; 50 μm in e, f; and 20 μm in g, h (*n* = 5. ***P < 0.001 vs control, in Student’s *t* test).

For successfully constructing a neuronal excitotoxicity model *in vivo*, KA (0.625 nmol) was injected into the striatum through stereotactic localization. On the 14th day after the intracranial injection, coronal sections of the brain were prepared for Nissl staining (Figure 1A). Representative images displayed that, neurons were hyperchromatic, pyknosis, and karyorrhexis after KA treatment (Figure 1B). Quantification of cells with normal morphology in the striatum showed that KA is significantly lethal to neurons (Figure 1C). In the subsequent experiments, we determined 0.625 nmol as the dosage in the model group.

**FIGURE 2:**
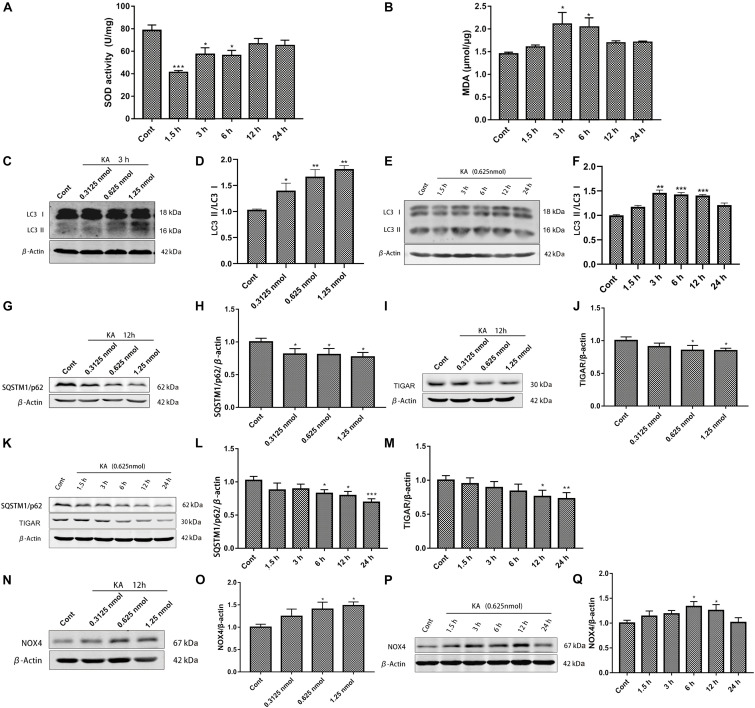
KA-induced excitotoxicity causes oxidative stress and autophagy activation. Mice were given different doses of KA or treated for different periods of time. **(A,B)** The time-course of total SOD activity and MDA content in the striatum. **(C–Q)** Representative bands and semi-quantitation of western blots for detecting LC3-II/LC3-I, SQSTM1/p62, TIGAR, and NOX4 protein levels. Data are expressed as the Mean ± SEM (*n* = 5. *P < 0.05, **P < 0.01, and ***P < 0.001 vs control, one-way ANOVA, followed by a *post hoc* multiple comparison Student-Newman–Keuls test).

The harmful consequences of glutamate receptor overactivation have been shown to be related to a series of events triggered by calcium-dependent enzyme activation, such as oxidative stress and mitochondrial damage (Divakaruni et al., 2017; Figures 2A,B). The significant changes of SOD activity and MDA content reflect the occurrence of oxidative stress. Interestingly, this change is short-lived. We hypothesized that it might be related to the increased nuclear translocation of Nrf2 that we detected (Tonelli et al., 2018), or due to compensatory stress.

We previously found that autophagy inhibitors partially blocked the toxicity of KA (Wang et al., 2008; Zhang et al., 2009). We examined the effect of KA on autophagy at different doses and treatment times. Increased ratio of LC3-II/LC3-I and decreased SQSTM1/p62 expression level embodied more autophagosomes and enhancive degradation, representing autophagy flows smoothly (Figures 2C–H,K,L; Kuma et al., 2017). TIGAR regulates the pathway of glycolysis. TIGAR can inhibit the occurrence of autophagy under nutritional starvation or metabolic stress. In the cellular antioxidant defense system, TIGAR may regulate autophagy as part of its constituent activity, which has a significant influence on the mTOR pathway (Geng et al., 2018; Zhang et al., 2019). It could not maintain the original expression level after KA treatment, which may exert a profound influence on autophagy (Figures 2I–K,M). KA injection into the striatum significantly increased NOX4 expression (Figures 2N–Q), but did not seem to affect NOX2 (Supplementary Figure 1).

There are still few suitable antioxidant drugs clinically available to deal with excitotoxicity in various neurodegenerative diseases. This study provides insights into seeking antioxidants to balance out excess ROS. So we focused on NADPH, which is known as coenzyme II and maintains the reduced GSH.

### NADPH Protects Neurons Against KA-Induced Excitotoxicity and Autophagy Activation

**FIGURE 3:**
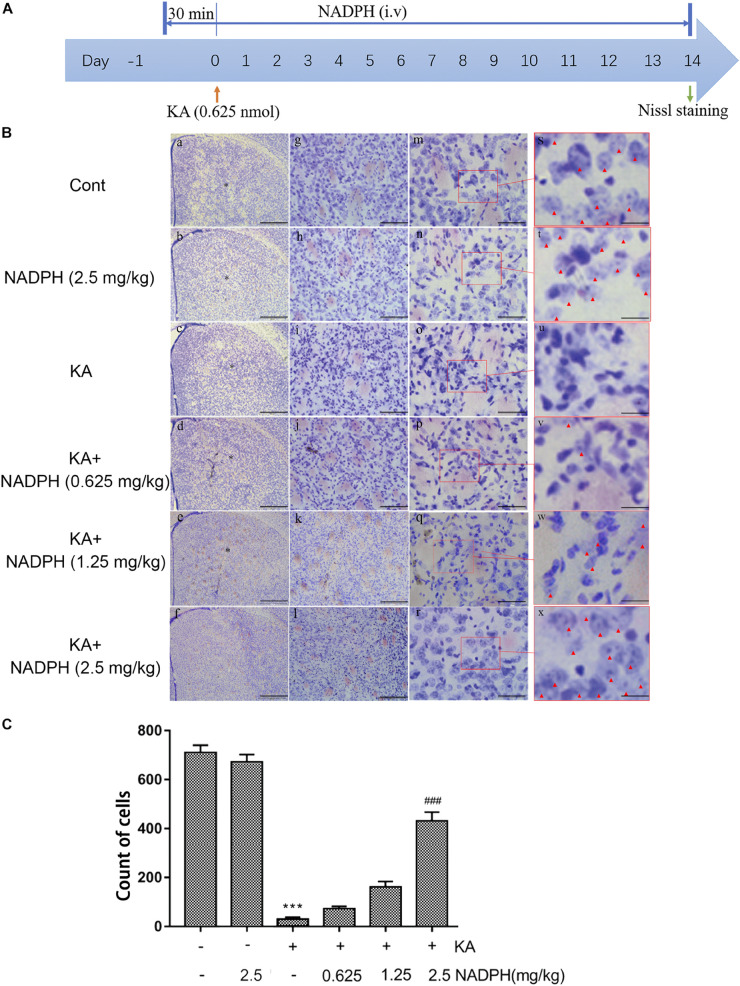
NADPH protects neurons against KA-induced excitotoxicity *in vivo*. **(A)** Illustration of experimental design. Mice were treated with 0.625, 1.25, and 2.5 mg/kg NADPH (*i.v*), 30 min before unilateral intrastriatal injection of 0.625 nmol KA. Administrate NADPH daily until mice were sacrificed 14 days later. Nissl staining were performed on brain sections. **(B)** Representative microscopic images of Nissl-stained coronal brain section displaying the lesion. **(C)** Quantification of neurons with normal morphological specificity. The histological analyses were single-blinded. Data are expressed as the Mean ± SEM. Scale bar = 500 μm in a–f; 100 μm in g–l; 50 μm in m–r; and 20 μm in s–x (*n* = 5. ***P < 0.001 vs control; ^###^*P* < 0.001 vs KA, one-way ANOVA, followed by a *post hoc* multiple comparison Student-Newman–Keuls test).

We investigated whether NADPH supplementation has an effect on KA-induced neuronal death and autophagy activation *in vivo* first. Mice were pretreated with NADPH (0.625, 1.25, and 2.5 mg/kg, *i.v*) 30 min before intrastriatal injection (Figure 3A). NADPH was then administered daily until day 14 through the tail vein. Pretreatment of 2.5 mg/kg NADPH notably reduced the nuclear condensation and abnormal neurons in the unilateral striatum (Figures 3B,C). Therefore, we performed the subsequent study with NADPH at 2.5 mg/kg.

**FIGURE 4:**
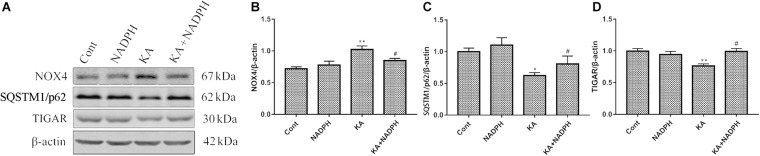
NADPH inhibits KA-induced autophagy activation and upregulation of NOX4. Animals were pre-treated with NADPH (2.5 mg/kg, *i.v*) 30 min prior to KA (0.625 nmol) injection and then they were sacrificed 12 h later for western blotting. **(A–D)** Representative bands and semi-quantitation of western blots for detecting NOX4, SQSTM1/p62 and TIGAR protein levels. Data are expressed as the Mean ± SEM (*n* = 5. *P < 0.05, **P < 0.01 vs control; ^#^*P* < 0.05 vs KA, one-way ANOVA, followed by a *post hoc* multiple comparison Student-Newman–Keuls test).

**FIGURE 5:**
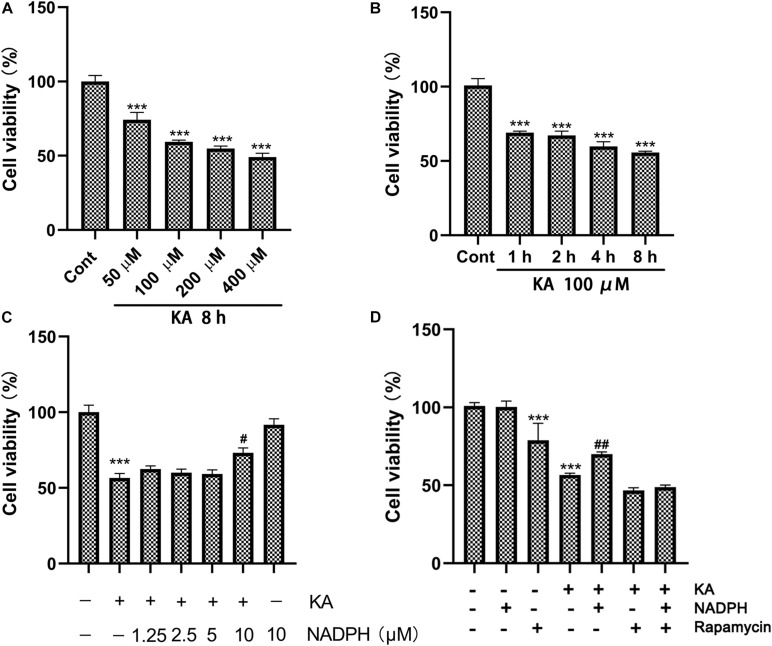
NADPH protects neurons against KA-induced excitotoxicity by sinhibiting autophagy *in vitro*. **(A,B)** Cultures were treated with indicated concentrations (100, 200, 400, and 800 μM) of KA for 8 h or KA (100 μM) for 1, 2, 4, and 8 h. **(C)** The effects of NADPH on KA-induced cytotoxicity. Cultures were pre-treated with NADPH (1.25, 2.5, 5, and 10 μM) for 4 h, then treated with KA (100 μM) or 8 h. **(D)** Cultures were pre-treated with NADPH (10 μM) and/or rapamycin (3 μM) for 4 h, then treated with KA (100 μM) for 8 h. Cell viability was measured by CCK8 kit. Data are expressed as the Mean ± SEM. (*n* = 5. ***P < 0.001 vs control; ^#^*P* < 0.05, ^##^*P* < 0.01 vs KA, one-way ANOVA, followed by a *post hoc* multiple comparison Student-Newman–Keuls test).

Western blot data showed that pre-treatment with NADPH reversed KA-induced upregulation of NOX4 and downregulation of SQSTM1/p62 and TIGAR protein levels (Figure 4). In the primary cortical neurons, NADPH treatment also has a certain significant effect (Figures 5A–C). We also found that rapamycin, an autophagy activator, was able to negate the protective effect of NADPH in primary cortical neurons (Figure 5D). These suggest that NADPH can inhibit KA-induced autophagy activation and that its protective effect is at least partially dependent on this mechanism.

Although NADPH has excellent curative effect, it has the defect of narrow therapeutic window, exposed in the experiment (Qin et al., 2017). This makes practical use and clinical research difficult. Considering its role in both ROS elimination and production, inhibition of NOX appears to be necessary for its future clinical application (Qin et al., 2017). Therefore, we set out to solve this problem.

### Mito-Apocynin Inhibits KA-Induced Upregulation of NOX4 on Mitochondria

Then we studied the effect of KA on NOX expression. NOX1, 2, and 4 are the main NOX subtypes expressed in neurons, while the role of NOX2 and NOX4 in neurodegenerative diseases has been relatively well studied (Ma et al., 2017). NADPH has a contradictory role in regulating redox. It can not only generate GSH to remove ROS, but also generate ROS as the substrate of NOX (Ying, 2008). Notably, the reversal of NOX4 expression level indicates that NADPH is less likely to produce ROS as its substrate, suggesting that the combination we are considering is meaningful. We studied the classic NOX inhibitors DPI and apocynin and developed two different combined administration regimens, which have been shown to have better effects when combined with NADPH, both for neuronal protection and motor function recovery, as shown in the (Supplementary Figures 2–5).

**FIGURE 6:**
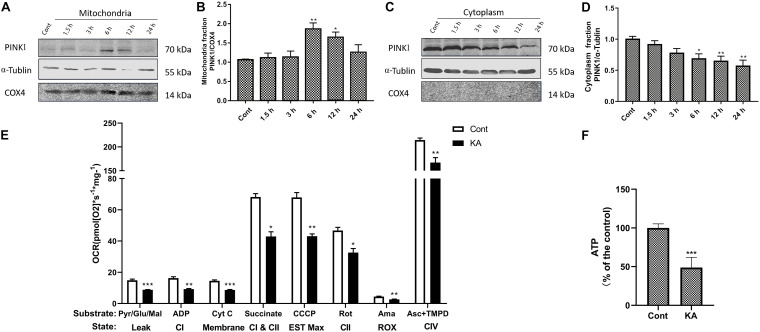
KA induces mitochondria dysfunction *in vivo*. **(A–D)** Mice were given different doses of KA or treated for different periods of time. Representative bands and semi-quantitation of western blots for detecting PINK1 protein levels in the mitochondria fraction and cytoplasm fraction. **(E)** Mitochondrial respiratory parameters determined by O2K. The control group and the KA group were labeled with white and black columns, respectively. **(F)** Quantification of ATP concentration. Data are expressed as the Mean ± SEM. Scale bar = 500 μm (*n* = 8. *P < 0.05, **P < 0.01, and ***P < 0.001 vs control, one-way ANOVA, followed by a *post hoc* multiple comparison Student-Newman–Keuls test).

**FIGURE 7:**
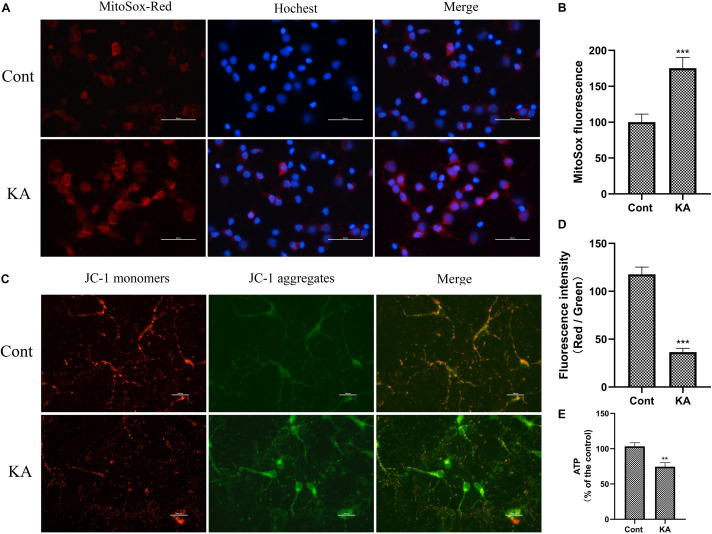
KA induces mitochondria dysfunction *in vitro*. **(A,B)** MtROS was detected and quantified by MitoSOX Red probe. **(C,D)** Mitochondrial membrane potential was examined and quantified by JC-1 staining assays. **(E)** Quantification of ATP concentration. Data are expressed as the Mean ± SEM. Scale bar = 50 μm (*n* = 8. **P < 0.01, ***P < 0.001 vs control, one-way ANOVA, followed by a *post hoc* multiple comparison Student-Newman–Keuls test).

Mitochondrial dysfunction is one of the important mechanisms of excitotoxicity leading to cell death (Wang et al., 2009). In depolarized mitochondria, PINK1 acted as a molecular sensor for damaged mitochondria, triggering the initiation of mitochondrial autophagy (Figures 6A–D). An important physiological function of PINK1 is to increase intracellular resistance to stress. Depletion of PINK1 in the cytoplasm increases the risk of stress-induced cell death. In cells deficient in PINK1, mitochondrial membrane proteins were reduced, affecting the efficiency of mitochondrial transport (Arena and Valente, 2017). The mitochondrial respiratory function and ATP-generating capacity in model group were significantly weaker than those in control group (Figures 6E,F). *In vitro*, KA resulted in decreased ATP level, loss of MMP and increased mitochondrial superoxide (Figure 7). Considering the regional limitations of NOX4 expression and mitochondrial dysfunction, mito-apocynin-C11, an inhibitor of mitochondrial NOX, was selected for combined treatment with NADPH.

**FIGURE 8:**
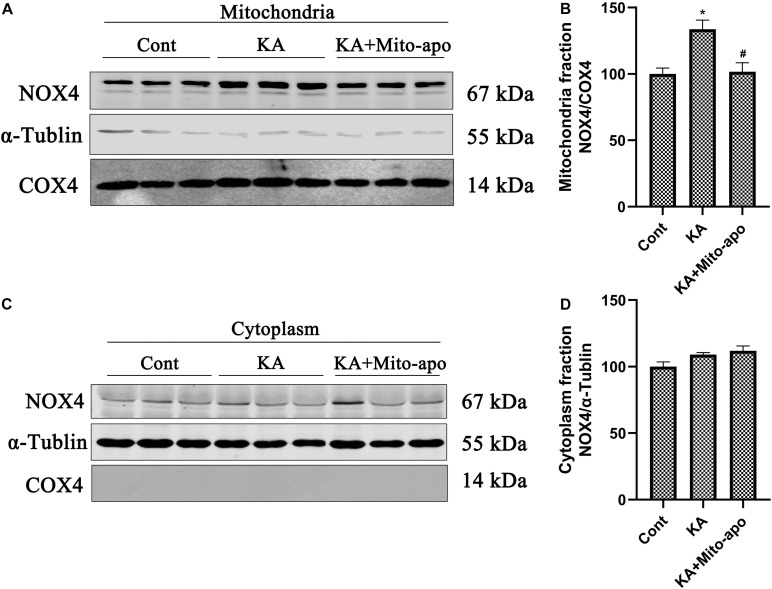
Mito-apocynin reverses KA-induced upregulation of NOX4 in mitochondria fraction. **(A–D)** Animals were pre-treated with mito-apocynin (displayed as Mito-apo in figures; 6 mg/kg, *i.g*) 1 day prior to KA (0.625 nmol) injection and then they were sacrificed 6 h later for western blotting. Representative bands and semi-quantitation of western blots for detecting NOX4 protein levels. Data are expressed as the Mean ± SEM (*n* = 5. *P < 0.05; ^#^*P* < 0.05 vs KA, one-way ANOVA, followed by a *post hoc* multiple comparison Student-Newman–Keuls test).

**FIGURE 9:**
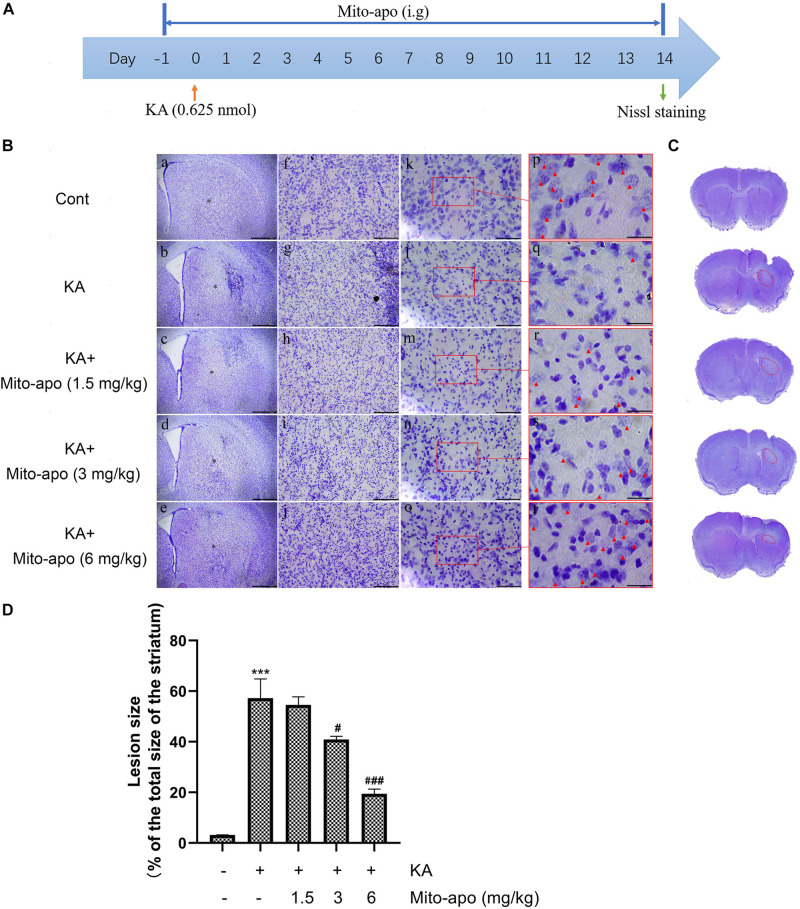
Effects of Mito-apocynin on KA-induced striatal neuronal death. **(A)** Illustration of experimental design. Mice were intragastric administrated with 1.5, 3, and 6 mg/kg Mito-apocynin, 1 day prior to KA (0.625 nmol) injection. Administrate Mito-apocynin daily until mice were sacrificed 14 days later. Nissl staining were performed on brain sections. **(B)** Representative microscopic images of Nissl-stained coronal brain section displaying the lesion. **(C)** Representative photographs of sections. The lession has been redlined. **(D)** Quantification of lesion area. Data are expressed as the Mean ± SEM. Scale bar = 500 μm in a–e; 100 μm in f–j; 50 μm in k-o; and 20 μm in p–t (*n* = 3. ***P < 0.001 vs control; ^#^*P* < 0.05, ^###^*P* < 0.001 vs KA, one-way ANOVA, followed by a *post hoc* multiple comparison Student-Newman–Keuls test).

Mito-apocynin-C11 apocynin conjugated to a mitochondria-targeting triphenyl phosphonium cation (TPP^+^) selectively target mitochondria NOXs via an alkyl chain consisting of eleven carbon atoms (Ghosh et al., 2016; Langley et al., 2017). The presence of a highly lipophilic and delocalized cationic moiety in Mito-apocynin-C11 makes it more cell-permeable and selectively target mitochondria (Dranka et al., 2014). Sequestration into mitochondria is facilitated by TPP^+^ conjugation to apocynin via long carbon–carbon side chains (Brenza et al., 2017). Mito-apocynin intragastric administration inhibits NOXs activity in the brain (Supplementary Figure 6). Further examination revealed that the change in NOX4 expression was mainly attributable to mitochondria, while the level in cytoplasm was relatively stable (Figure 8). Mito-apocynin can improve neuronal survival and indirectly inhibit mitochondrial NOX expression (Figures 8,  9).

### Combined NADPH and Mito-Apocynin Provides Greater Neuroprotective Effects and Motor Recovery

**FIGURE 10:**
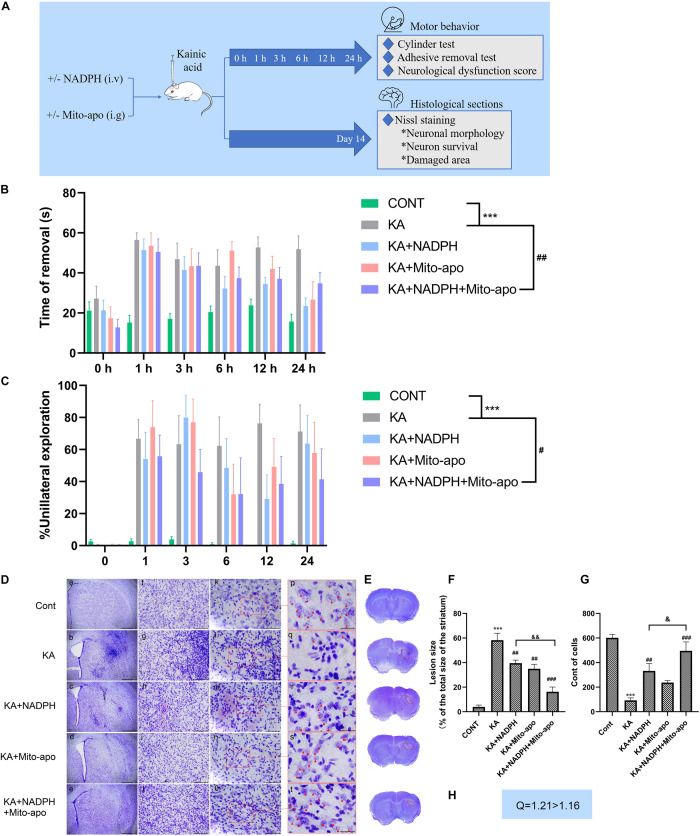
Combined NADPH and Mito-apocynin provides greater motor recovery and neuroprotective effects. **(A)** Illustration of experimental design. Mice were treated with NADPH and/or Mito-apocynin before KA injection. Behavioral tests were performed at 1, 3, 6, 12, and 24 h. The mice were sacrificed on the 14th day to prepare histological sections for Nissl staining. **(B)** Adhesive removal test. **(C)** Cylinder test. **(D)** Representative microscopic images of Nissl-stained coronal brain section displaying the lesion. **(E)** Representative photographs of sections. The lesion has been redlined. **(F)** Quantification of lesion area. **(G)** Quantification of neurons with normal morphological specificity. **(H)** Combination index. Data are expressed as the Mean ± SEM. Scale bar = 500 μm in a–e; 100 μm in f–j; 50 μm in k–o; and 20 μm in p–t (*n* = 6. ***P < 0.001 vs control; ^#^*P* < 0.05, ^##^*P* < 0.01, ^###^*P* < 0.001 vs KA; ^&^*P* < 0.05, ^&&^*P* < 0.01 KA + NADPH vs KA + NADPH + Mito-apocynin, one-way ANOVA, followed by a *post hoc* multiple comparison Student-Newman–Keuls test).

To determine whether combined Mito-apocynin can enhance the neuroprotective effects of NADPH, we chose their minimum effective dose for further study *in vivo*. The design of the experiment can be seen in Figure 10A.

Lesions in the striatum can lead to impaired voluntary movement and muscle tone, especially in the forelimbs (Hidalgo-Balbuena et al., 2019). So we detect impaired motor function as an indicator of neural dysfunction due to excitotoxicity, by performing the adhesive removal test and the cylinder test of forelimb asymmetry (Hernández-Espinosa et al., 2019). Before stereotactic KA injection, the mice could quickly remove the adhesive label and gave priority to touch (Hernández-Espinosa et al., 2019) the container wall with both forelimbs. KA resulted in a significant delay to remove and reduction of bilateral touch in each group. Compared to NADPH, combination therapy group showed similar impaired motor function at 1 h, but better recovery in the later with statistical significance (Figures 10B,C). Using NADPH alone, the morphology striatal neurons were not significantly restored as in combination therapy group. Besides, combination administration shrunk the lesion area and increase cell count compared to the monotherapy (Figures 10D—H).

The present findings confirm that combination of NADPH and Mito-apocynin can significantly improve the neuronal survival in the neuronal excitotoxicity model, and enhance the rehabilitation of neurobehavioral defects, by speeding up the recovery of muscle tone, fine motor ability and limb coordination. Then we further investigated whether this better effect was associated with further increase in GSH and inhibition of autophagy.

### Combined NADPH and Mito-Apocynin Further Increased GSH Levels and Inhibited Autophagy

**FIGURE 11:**
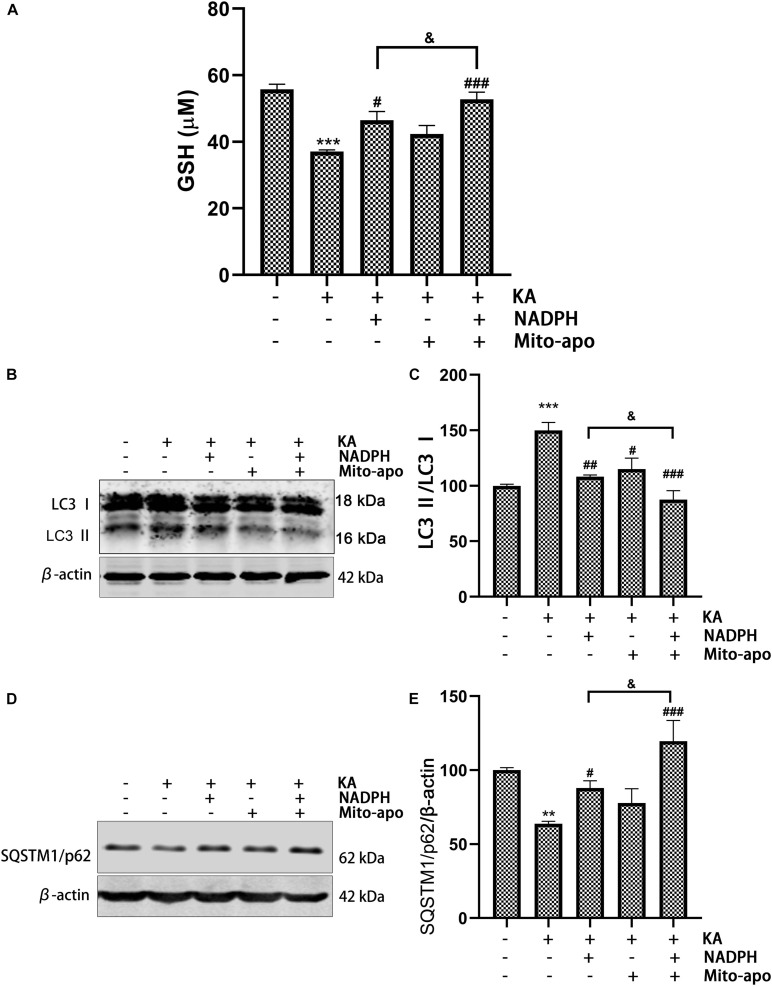
Combined NADPH and Mito-apocynin further increases GSH levels and inhibited autophagy. Animals were pre-treated with Mito-apocynin (3 mg/kg, *i.g*) and/or NADPH (2.5 mg/kg, *i.v*) prior to KA (0.625 nmol) injection. **(A)** They were sacrificed 12 h later for determination of GSH. **(B,C)** They were sacrificed 3 h later. Representative bands and semi-quantitation of western blots for detecting LC3 protein levels. **(D,E)** They were sacrificed 12 h later. Representative bands and semi-quantitation of western blots for detecting SQSTM1/p62 protein levels. Data are expressed as the Mean ± SEM. (*n* = 5. **P < 0.01, ***P < 0.001 vs control; ^#^*P* < 0.05, ^##^*P* < 0.01, and ^###^*P* < 0.001 vs KA; ^&^*P* < 0.05, KA + NADPH vs KA + NADPH + Mito-apocynin, one-way ANOVA, followed by a *post hoc* multiple comparison Student-Newman–Keuls test).

**FIGURE 12:**
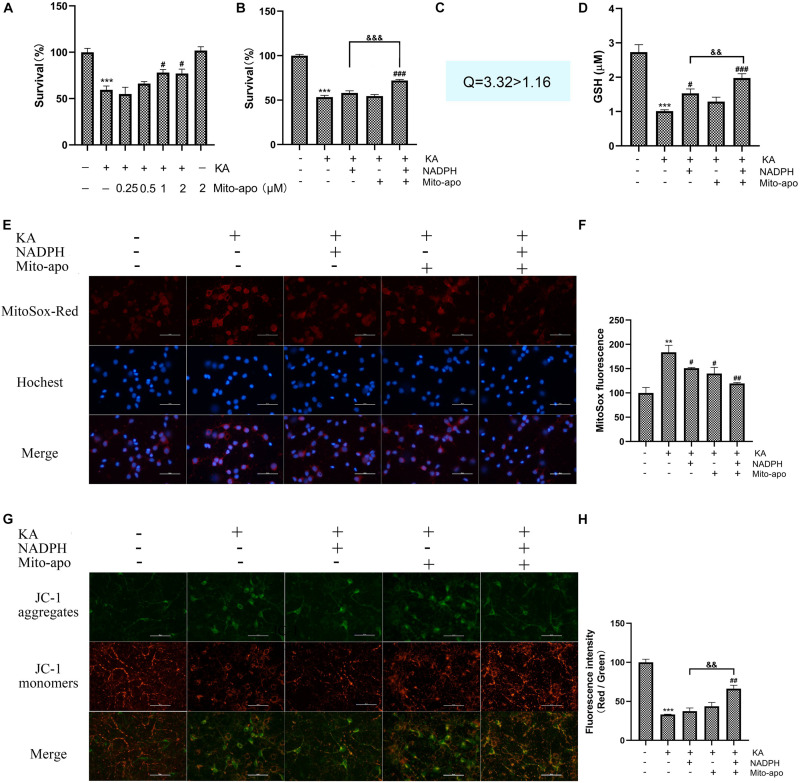
Effects of combined NADPH and Mito-apocynin on KA-induced mitochondria dysfunction *in vitro*. Cultures were pre-treated with NADPH and/or mito-apocynin for 4 h, then treated with KA (100 μM) for 8 h. **(A,B)** Cell survival detected by CCK-8 kit. C_*NADPH*_ = 5 μM; C_*mito–apo*_ = 0.5 μM. **(C)** Combination index. C_*NADPH*_ = 5 μM; C_*mito–apo*_ = 0.5 μM. **(D)** Determination of GSH. C_*NADPH*_ = 10 μM; C_*mito–apo*_ = 1 μM. **(E,F)** MtROS was detected and quantified by MitoSOX Red probe. C_*NADPH*_ = 5 μM; C_*mito–apo*_ = 0.5 μM. **(G,H)** Mitochondrial membrane potential was examined and quantified by JC-1 staining assays. C_*NADPH*_ = 5 μM; C_*mito–apo*_ = 0.5 μM. Data are expressed as the Mean ± SEM. Scale bar = 50 μm (*n* = 5. **P < 0.01, ***P < 0.001 vs control; ^#^*P* < 0.05, ^##^*P* < 0.01, and ^###^*P* < 0.001 vs KA; ^&&^*P* < 0.01, ^&&&^*P* < 0.001, KA + NADPH vs KA + NADPH + Mito-apocynin, one-way ANOVA, followed by a *post hoc* multiple comparison Student-Newman–Keuls test).

.5 NADPH can restore KA-induced GSH level decline *in vivo* and *in vitro*, and play an antioxidant role. In combination, GSH content increases further (Figures 11A, 12D). This indicates that NADPH does, as expected, reduce the proportion of ROS production as a substrate of NOX, and tends to be a reducing agent. In addition, NADPH further reduced LC3-II/LC3-I and increased SQSTM1/p62 expression when combined with Mito-apocynin (Figures 11B–E). Mito-apocynin appears to enhance the inhibition of autophagy by NADPH. We concluded that combination of NADPH and mito-apocynin provides better neuroprotection and motor recovery through stronger antioxidant and autophagy balance.

### Effects of Combined NADPH and Mito-Apocynin on KA-Induced Mitochondria Dysfunction *in vitro*

As mentioned above, we found the phenomena of increased mitochondrial superoxide anions and decreased membrane potential. We preliminarily investigated the effect of combination on mitochondrial dysfunction. Pretreatment with 1 μM Mito-apocynin (Figure 12A) or 10 μM NADPH for 4 h significantly improved neuronal survival. When both ineffective dose of NADPH and Mito-apocynin were pretreated, the combination group showed a synergistic effect, as evidenced by combination index (Figures 12B,C).

In terms of reducing mitochondrial superoxide production, syndication is not so superior (Figures 12E,F). In terms of restoring MMP, combined administration is significantly better than monotherapy, (Figures 12G,H). The results show that NADPH play a role in reducing mitochondrial dysfunction and, in some ways, combining mito-apocynin is better. Further studies on NADPH and mitochondria are needed.

## Discussion

Overstimulation of glutamate receptors increases the production of free radicals, leading to the development of a variety of neurodegenerative diseases (Serwach and Gruszczynska-Biegala, 2019). Some researchers recognize the efficacy of antioxidants on animal models of Huntington’s diseases, Parkinson’s diseases, Alzheimer’s diseases, and stroke (Hughes et al., 2016; Huang et al., 2018; Pinho et al., 2020). In this study, we stimulate glutamate receptors specifically and efficiently to construct representative neurodegenerative disease models, by precisely injecting kainic acid (KA) into the striatum. It mediates ROS accumulation *in vivo*, which can be fatal to neurons. Exogenous NADPH and Mito-apocynin, a mitochondrial targeted NOX inhibitor, both offer efficacy against excitotoxic injury to some extent. The mechanisms by which they operate are associated with maintaining reductive substances and reducing the generation of free radicals. Our previous studies have shown that systemic administration of NADPH can penetrate the blood-brain barrier (Li et al., 2016). Intracellular NADPH concentration in cultured mouse cortical neurons was significantly increased after the addition of exogenous NADPH. The mechanism of NADPH transmission through the membrane had been a largely under explored domain. We have preliminary research on this issue, but more exploration is needed.

The upregulation of NOX4 expression in striatum, especially in mitochondria, increases the risk of oxidative stress. NADPH could inhibit KA-mediated upregulation of NOX4 expression, indicating that the possibility of ROS generation by NADPH was decreased. Therefore, it is of great significance to study whether the combination of NADPH and NOX inhibitors can play a better neuroprotective role in neuroexcitatory injury. Two NOX inhibitors (DPI and apocynin), two combined schemes (combination of ineffective or effective dose), fully demonstrate the advantages of combined use, both in terms of reducing injury and restoring motor function. Mito-apocynin was selected because of occurrence of NOX4 regional elevations and mitochondrial dysfunction. Its presence greatly increases the potency of NADPH by further increasing the content of GSH that can be produced. Lesions and neuronal death were greatly reduced in the striatum. While any monotherapy did not significantly improve the neurobehavioral of mice, the combination therapy did.

Under some pathological conditions, such as stroke and neurodegenerative diseases, relatively excessive ROS accumulation will destroy cell homeostasis, leading to oxidative stress and mitochondrial dysfunction, and induce autophagy. In this process, oxidative stress promotes autophagy. In turn, autophagy helps to reduce oxidative damage by engulfing and degrading oxidizing substances (Scherz-Shouval and Elazar, 2011). The internal regulation mechanism of ROS autophagy includes various molecular signaling pathways, such as ROS-FOXO3-LC3/BNIP3-autophagy, ROS-NRF2- SQSTM1/p62-autophagy, and ROS-TIGAR-autophagy (Li et al., 2015). NADPH inhibits overactive autophagy caused by excessive ROS in excitotoxicity. And its protective effect can be weakened by autophagy activators. Its dual effect on ROS limits its ability to restore autophagy balance. This drawback seems to be alleviated through a combination of mito-apocynin.

Excitotoxicity resulted in significant mitochondrial impairment *in vitro* and *in vivo*, including enhanced PINK1 signal, increased superoxide, decreased MMP, and reduced ATP production. Antioxidant strategies are abundant, but they do not perform well in neurodegenerative diseases. Mitochondria is recognized as one of the sources of ROS (Dan Dunn et al., 2015). Recent data support that mitochondrial targeted NOX inhibitors have a certain effect on PD, but there are few studies on its mechanism (Dranka et al., 2014; Langley et al., 2017). Mito-apocynin was effective in narrowing the lesion and had a synergistic amplification effect with NADPH. It may reduce NADPH as the substrate of mitochondrial NOX and increase superoxide anions, showing less damage and better movement recovery. The concomitant consequences of excitotoxicity may be in large part related to mitochondrial dysfunction caused by calcium stress. 5 μM NADPH and 0.5 μM mito-apocynin were not significant on the cell activity test. They failed to restore MMP, but had a significant effect when combined. But no significant difference has been found in MtROS reduction.

**FIGURE 13:**
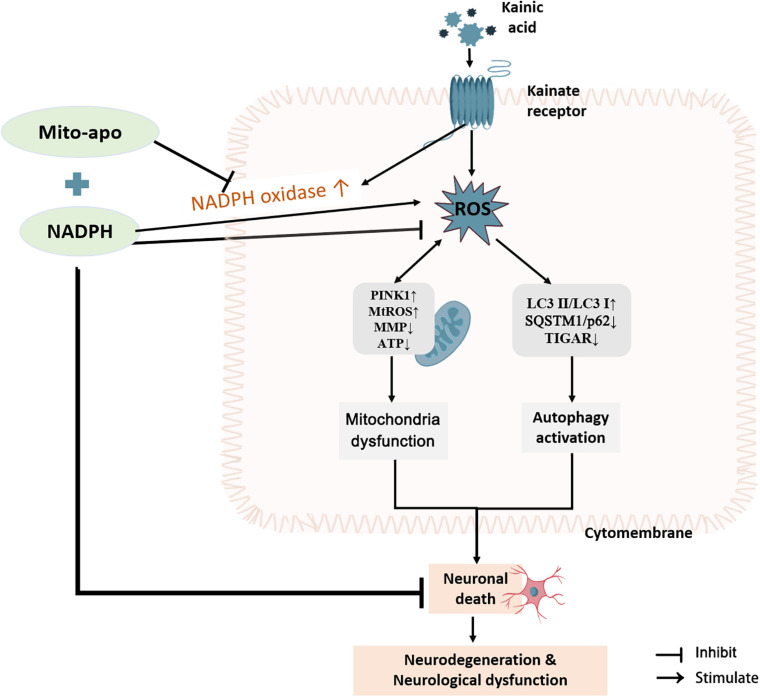
Combination of NADPH and Mito-apocynin offers greater neuroprotection and resistance to mitochondrial impairment on KA-induced excitotoxicity. KA induces the increase of ROS in the striatum, following by autophagy activation and mitochondrial stress, eventually leading to neuronal death, and subsequent neurodegeneration and neurological dysfunction. NADPH can improve neuron survival by reversing the protein expression of SQSTM1/p62 and TIGAR and sheltering mitochondria. When combined with Mito-apocynin, it reduces NADPH as a NOX substrate to generate ROS, and finally performs better neuroprotective effects and resistance to mitochondrial impairment.

Collectively, NADPH protects neurons from excitotoxic damage by blocking autophagy activation through antioxidant activity. When used in combination with NOX inhibitors (DPI, apocynin, and Mito-apocynin), the neuronal protection and mobility restoration capabilities are even more powerful. The reason may be the reduction of NADPH as a substrate to promote ROS during the reduction stress. When the NOX inhibitor was targeted at mitochondria, it has an extra miraculous effect on MMP resumption. Maintenance of mitochondrial function may be considered a promising aspect of resisting excitotoxicity (Figure 13).

## Data Availability Statement

The raw data supporting the conclusions of this article will be made available by the authors, without undue reservation.

## Ethics Statement

The animal study was reviewed and approved by The Ethical Committee of Soochow University.

## Author Contributions

YW conceived and designed the research. NL, M-ML, S-SH, and Z-QL collected data and conducted the research. NL wrote the initial manuscript. YW, J-CW, Z-QL, and Z-HQ revised the manuscript. All authors read and approved the final manuscript.

## Conflict of Interest

The authors declare that the research was conducted in the absence of any commercial or financial relationships that could be construed as a potential conflict of interest.
