# Supplementary material for: NADPH and Mito-Apocynin Treatment Protects Against KA-Induced Excitotoxic Injury Through Autophagy Pathway
Source: Front Cell Dev Biol. 2021 Feb 11;9:612554. doi: 10.3389/fcell.2021.612554 (PMC7905037; doi:10.3389/fcell.2021.612554)
Supplement: Supplementary file 1 [file Table_1.pdf]

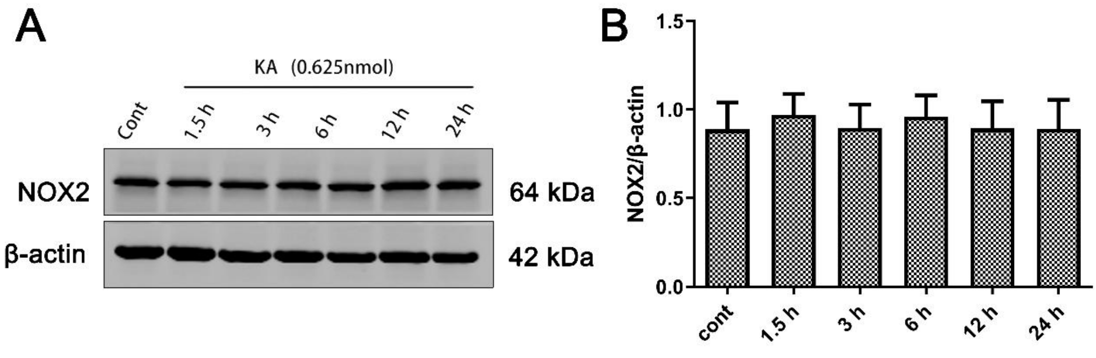

Fig. S1 KA did not cause changes in NOX2 expression. (A，B) Representative bands and semi-quantitation of western blots for detecting NOX2 protein levels. Data are expressed as the Mean ± SEM (n = 6. one-way ANOVA, followed by a post hoc multiple comparison Student-Newman-Keuls test).

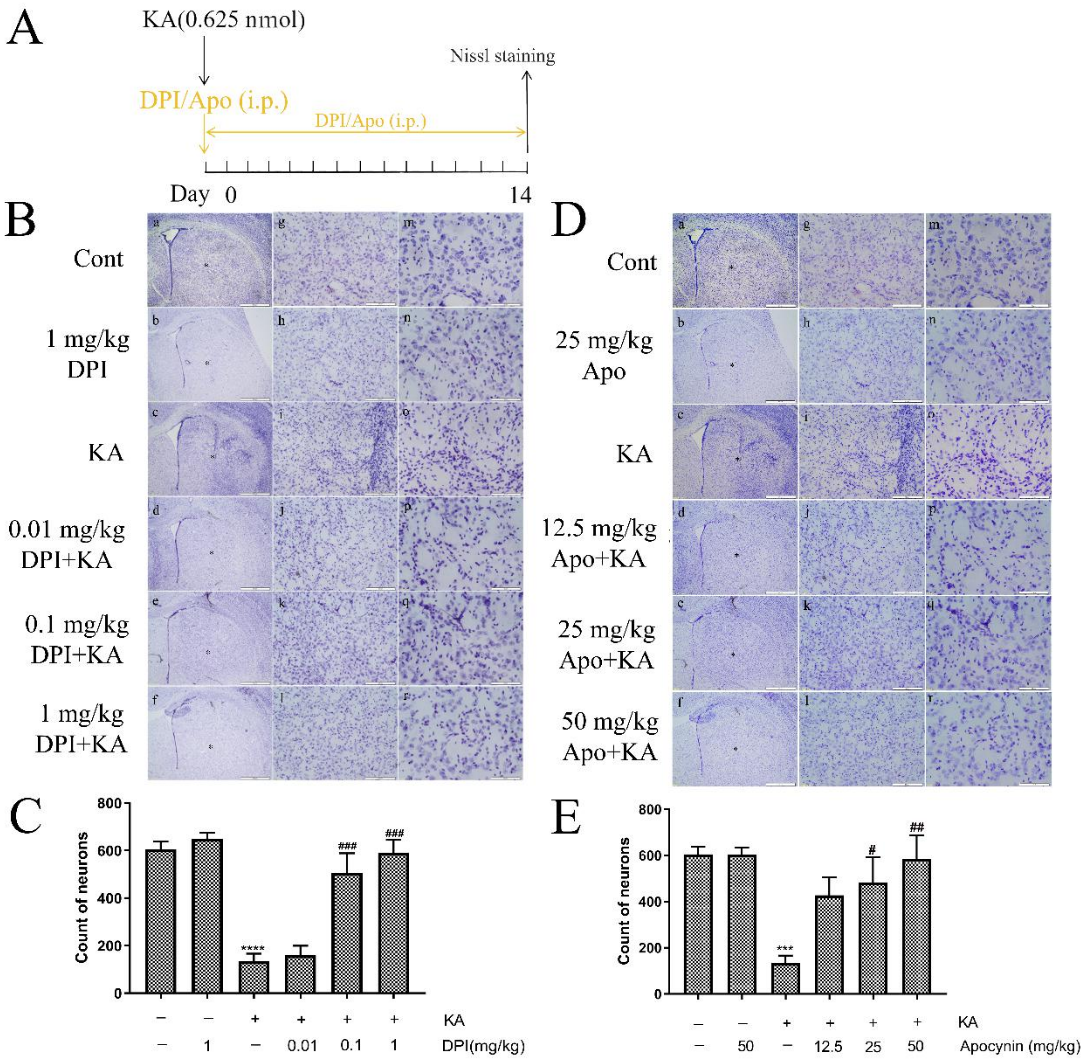

Fig. S2 Effects of NOX inhibitors on KA-induced neuronal death. (A) Illustration of experimental design. Mice were treated with 0.01, 0.1, 1 mg/kg DPI or 12.5, 25, 50 mg/kg apocynin via intraperitoneal injection 30 min before unilateral intrastriatal injection of 0.625 nmol KA, dosing once a day for two weeks.

Paraformaldehyde-fixed brain sections were stained with Nissl staining solution. **(B, D)** Representative micrographs were taken in the center of drug injection (adjacent to needle tracks). **(C, E)** Quantitative results of neurons with normal morphological characteristics in central striatum. The counting was done by another researcher in a randomized, single-blind manner. Data were expressed as the Mean ± SEM. Scale bar = 500 μm in a-f; 100 μm in g-l; 50 μm in m-r (n = 5. *** P < 0.001 vs control; ### P < 0.001 vs KA, one-way ANOVA followed by Student- Newman-Keuls post hoc test).

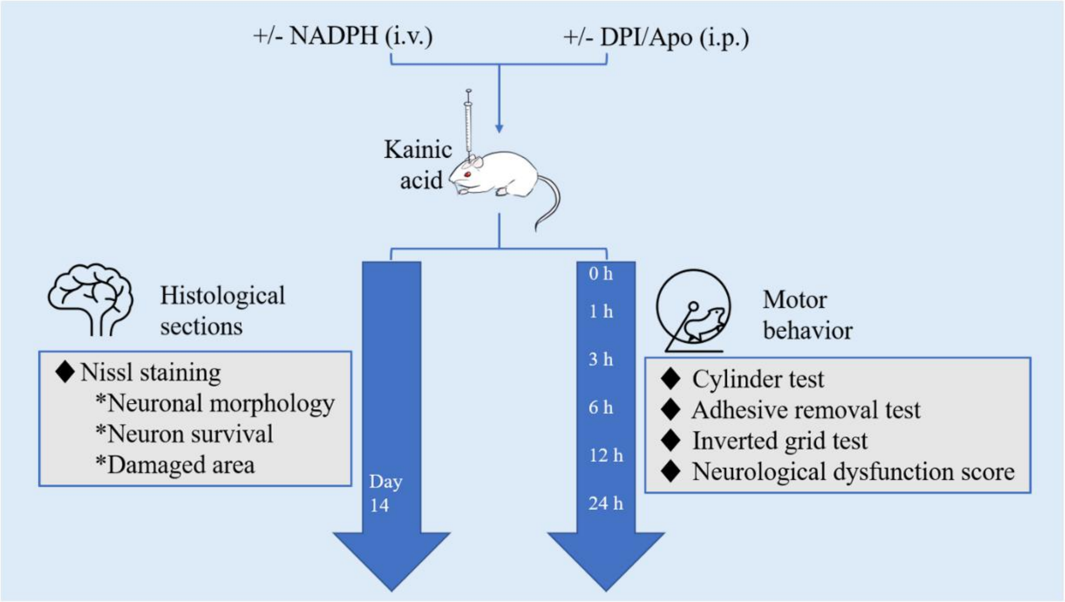

Fig. S3 Illustration of experimental design. Mice were treated with NADPH and/or NOX inhibitors 30 mins before KA injection. Behavioral tests were performed at 1, 3, 6, 12 and 24 h. The mice were sacrificed on the 14th day to prepare histological sections for Nissl staining.

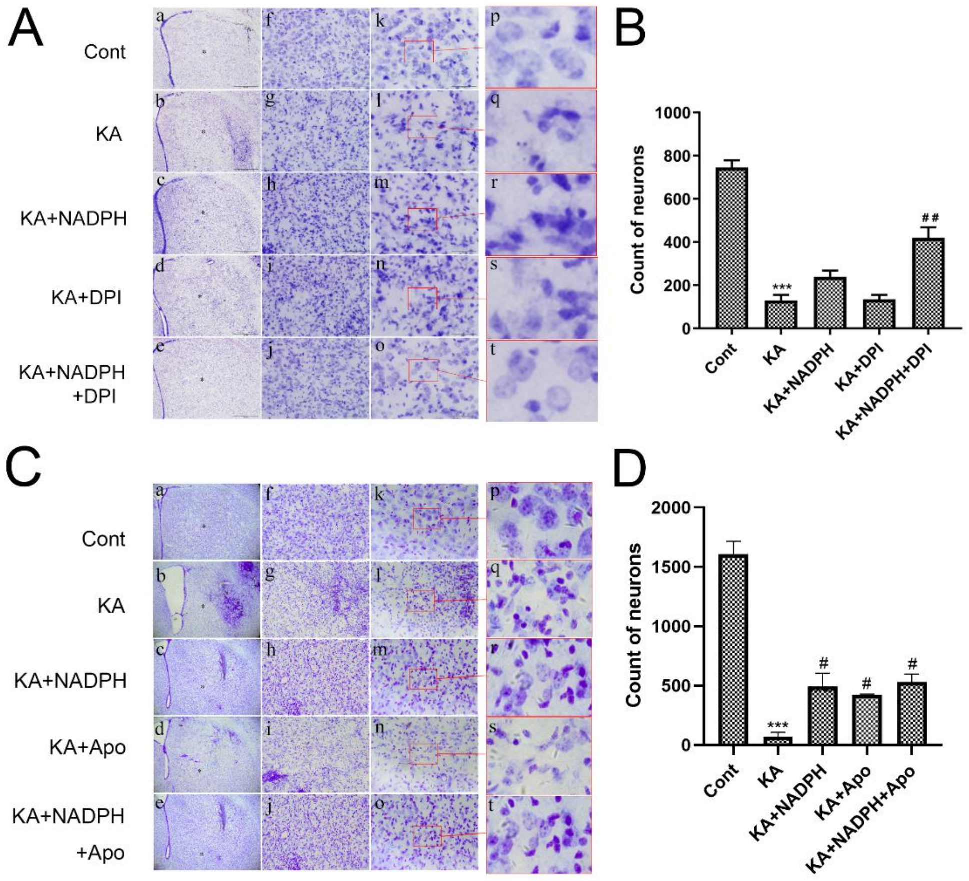

Fig. S4 Combined NADPH and NOX inhibitors provides greater neuroprotective effects. Animals were administered NADPH (1.25 mg/kg) and/or DPI (0.01 mg/kg) in A-B. Animals were administered NADPH (2.5 mg/kg) and/or apocynin (25 mg/kg) in C-D. Then they were sacrificed after 14 days. Paraformaldehyde-fixed brain sections were stained with Nissl staining solution. **(A, C)** Representative micrographs were taken in the center of drug injection (adjacent to needle tracks). **(B, D)** Quantitative results of neurons with normal morphological characteristics in central striatum. The counting was done by another researcher in a randomized, single-blind manner. Data were expressed as the Mean ± SEM. 500 µm in a-e; 100 µm in f-j; 50 µm in k-o(n = 5. *** $P < 0.001$ vs control; ## $P < 0.01$ vs KA, one-way ANOVA followed by Student- Newman-Keuls post hoc test).

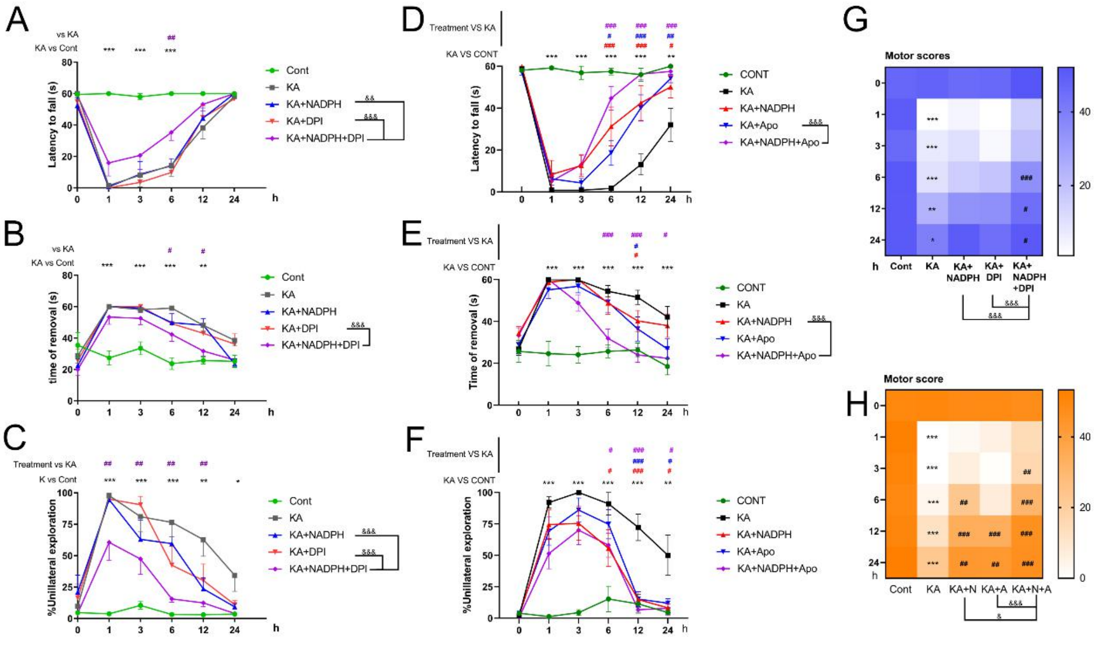

Fig. S5 Combined NADPH and NOX inhibitors provides greater motor recovery. Animals were administered NADPH (1.25 mg/kg) and/or DPI (0.01 mg/kg) in A-B. Animals were administered NADPH (2.5 mg/kg) and/or apocynin (25 mg/kg) in C-D. Performance was evaluated in three tests of motor behavior at 1, 3, 6, 12, and 24 h after KA intracerebral injection. These tests were later integrated to obtain a motor score that reflects functional deterioration. **(A, D)** Inverted grid test. **(B, E)** Adhesive removal test. **(C, F)** Cylinder test. **(G, H)** To integrate the three previous tests, an arbitrary score was assigned to the performance of each animal, giving the highest score to the performance of the mice injected with saline (60 points). Data were expressed as the Mean ± SEM (n = 8. * P < 0.05, ** P < 0.01, *** P < 0.001 vs control; # P < 0.05, ## P < 0.01, ### P < 0.001 vs KA; & < 0.05, && < 0.01, &&& < 0.001 vs monotherapy, one-way ANOVA followed by Student-Newman- Keuls post hoc test).

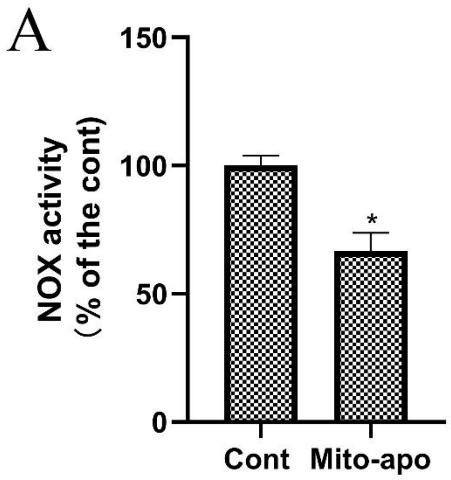

Fig. S6 Oral administration of Mito apocynin can inhibit the activity of NOXs in brain. Data are expressed as the Mean ± SEM (n =3. one-way ANOVA, followed by a post hoc multiple comparison Student-Newman-Keuls test).
